# Development and validation of the Meiji Nutritional Profiling System for children

**DOI:** 10.3389/fnut.2025.1611286

**Published:** 2025-06-17

**Authors:** Ryota Wakayama, Tao Yu, Adam Drewnowski, Satoshi Takasugi, Tomohito Horimoto, Chiharu Tsutsumi

**Affiliations:** ^1^Meiji Co., Ltd., Chuo City, Tokyo, Japan; ^2^Center for Public Health Nutrition, University of Washington, Seattle, WA, United States; ^3^Department of Nutrition and Health, Faculty of Nutritional Science, Sagami Women's University, Sagamihara, Kanagawa, Japan

**Keywords:** nutrient profiling, World Health Organization, nutrient-rich food index, growth, development, children, Japanese diet

## Abstract

**Background:**

The Meiji Nutritional Profiling System (Meiji NPS) takes into account life-stage differences and addresses specific health challenges in different age groups in Japan.

**Objectives:**

This study aimed to develop the Meiji NPS for younger (3–5 years) and older (6–11 years) children to encourage product reformulation and promote proper growth and development.

**Methods:**

Meiji NPS scores for 1,091 foods listed in the Japanese Food Standard Composition Table were calculated and compared with nutrient profiles (NPs) for children developed by the WHO Regional Office for the Western Pacific or Nutrient-Rich Foods Index 9.3 (NRF9.3).

**Results:**

For younger children, the Meiji NPS scores ranged from −442.3 to 423.9, and for older children, the Meiji NPS scores ranged from −265.5 to 386.5. The Meiji NPS scores showed significant differences (*p* < 0.001) between healthy and unhealthy foods, when classified according to the WHO NP model. There was also a strong correlation between the Meiji NPS and NRF9.3, validating the new NPS system (*r* = 0.73).

**Conclusion:**

The Meiji NPS for younger and older children may provide a useful incentive for Japanese food manufacturers to produce healthier products.

## 1 Introduction

The World Health Organization (WHO) defines nutrient profiling (NP) as the science of classifying foods according to their nutritional composition (1). NP models are designed to capture the overall nutrient density of foods and are generally expressed in terms of nutrients per 100 kcal, 100 g, or per serving size (2). The selection of specific index nutrients is based on the health concerns and nutrient needs of the population (3–7). The main purpose of NP models is to improve diet quality, a vital component of health promotion and disease prevention (1).

The WHO specifies that NP models should address identified challenges in population health (1). One such challenge is the increasing prevalence of over-nutrition, which leads to obesity and overweight (8). Most NP models, including the one developed by the WHO for the Western Pacific region (9), penalize calories, added sugars, saturated fats, and salt. However, inadequate nutrition is still prevalent and can vary according to life stage. The Health Japan 21 report has emphasized the importance of a life-course approach for the Japanese population (10). Nutrition-related health challenges in Japan include inadequate nutrition among children, lifestyle-related diseases and thinness among young women (11–22), and nutrient deficits and frailty among older adults (23, 24).

The Meiji Nutritional Profiling System (NPS) is sensitive to life-stage differences in Japan (25–27). While the Meiji NPS for adults (≥12 years) and older adults (≥65 years) has already been developed, there is currently no NPS that addresses children's nutritional and health needs. Two health-related priorities were identified for younger (aged 3–5 years) and older (aged 6–11 years) children: ensuring adequate nutrition for optimal growth and development, which is crucial, and preventing childhood overweight and obesity, which are risk factors for future lifestyle-related diseases (10, 28–33). The proposed approach is to address health issues by creating NP models that are specific to each life stage.

Whereas many NP models, including the WHO model, are designed for consumer use, NP modeling can also provide value to the food industry. NP models have been used to support the front-of-pack (FOP) labels designed to steer children away from unhealthy foods (9, 34). By focusing on excess calories and nutrients to limit, these NP models justify restrictions on marketing and advertising targeted at children (5, 35). Nutrient profiling can also serve a complementary purpose, which may be even more valuable for global public health. Food manufacturers have used NP models to guide and benchmark product reformulation and to support the development of nutrient-rich foods (36–40). Product reformulation is the most valuable application of NP models in the food industry.

The present age-specific approach was used to develop a Meiji NPS for younger and older children, based on their nutrition needs. The Meiji NPS framework would then provide scientific support for product reformulation for each age group in Japan and thus improve the quality of the food supply. To the best of our knowledge, no previous NPS had listed the reformulation of food products for Japanese children as its main goal. This study focused on childhood nutrition to support optimal growth and development, as predictors of future good health. Food manufacturers may identify the Meiji NPS framework useful to develop healthier products.

## 2 Materials and methods

### 2.1 Scope of the Meiji NPS for children

The present study aimed to develop a version of the Meiji NPS that is age-sensitive and specifically tailored to the nutritional needs of younger (ages 3–5 years) and older (ages 6–11 years) children. The previously published (25) the Meiji NPS for adults and older adults is based on nutrients to encourage, food groups to encourage, and nutrients to limit. The ratios of each component were calculated relative to age-appropriate reference daily values (RDVs) (25). Consistent with the Nutri-Score and Health Star Rating, the Meiji NPS includes energy among the elements to be limited.

### 2.2 Selection of nutrients and food groups for the Meiji NPS for children

The new Meiji NPS for children was also based on nutrients to encourage, food groups to encourage, and nutrients to limit. Nutrients to encourage were protein, dietary fiber, calcium, iron, and vitamin D (25). These nutrients are important for children's growth and development (41–48) but are consumed by children in inadequate amounts, according to the Dietary Reference Intakes for Japan (2020) (11) and the 2019 National Health and Nutrition Survey (49). Nutrients to limit were energy, saturated fatty acids (SFAs), sugar (the sum of glucose, galactose, fructose, maltose, sucrose, and lactose), and salt equivalents. The WHO NP model included similar nutrients to limit (9). The food groups to encourage included dairy, fruits, vegetables, nuts, and legumes (25, 50). In Japan, school lunches provide balanced meals supplying the necessary nutrients (51, 52). However, given that there are ~160 school holidays in a year (53), the days without school lunches may adversely affect children's overall diet quality.

### 2.3 Algorithms of the Meiji NPS for children

The Meiji NPS is based on the Nutrient-Rich Foods Index 9.3 (NRF9.3), a well-established and validated NP model (54–56). The Meiji NPS has also been validated based on convergent and predictive validity in the Japanese population (25–27). The algorithms of the Meiji NPS for children are based on both NRF9.3 and the Meiji NPS for adults. The Meiji NPS for children calculates scores based on the ratios of nutrients to encourage, nutrients to limit, and food groups to encourage, relative to their RDVs per 100 g or per serving size (Equations 1, 2).


(1) $$\begin{array}{l} Meiji\;NPS\;for\;younger\;children\\ =\sum_{i=1}^{5}\left(\frac{{nutrients\;to\;encourage}_{i}}{RDV_{i}}\right)\times 100\\ -\sum_{i=1}^{4}\left(\frac{{nutrients\;to\;limit}_{i}}{RDV_{i}}\right)\times 100\\ +\sum_{i=1}^{5}\left(\frac{{food\;groups\;to\;encourage}_{i}}{RDV_{i}}\right)\times 100 \end{array}$$

(2) $$\begin{array}{l} Meiji\;NPS\;for\;older\;children\\ =\sum_{i=1}^{5}\left(\frac{{nutrients\;to\;encourage}_{i}}{RDV_{i}}\right)\times 100\\ -\sum_{i=1}^{4}\left(\frac{{nutrients\;to\;limit}_{i}}{RDV_{i}}\right)\times 100\\ -\sum_{i=1}^{5}\left(\frac{{food\;groups\;to\;encourage}_{i}}{RDV_{i}}\right)\times 100 \end{array}$$

### 2.4 Age-appropriate RDVs

The Meiji NPS calculations per 100 g and serving size (25, 26) used RDVs for nutrients to encourage and those to limit. The RDVs were obtained from the Dietary Reference Intakes for Japan (11) and by the WHO recommendations (57, 58). RDVs for food groups to encourage in the Meiji NPS for adults were based on Health Japan 21 (59) and EAT-Lancet planetary health diet guidelines (60). For children, the current RDVs were calculated by adjusting the RDV of the energy set in the Meiji NPS for adults according to the children's energy needs. Specifically, the ratios were 46% for younger children (1,300 kcal/2,800 kcal, 46%) and 80% for older children (2,250 kcal/2,800 kcal, 80%). A 100% cap was not applied for nutrients to limit, consistent with the WHO NP model, which prioritizes nutrients to limit. The age-appropriate nutrient standards and 100% RDV caps are summarized in Table 1.

**Table 1:** RDVs and caps of the Meiji NPS for younger children and older children.

**Items**		**Meiji NPS for younger children**		**Meiji NPS for older children**	
		**RDV**	**Cap**	**RDV**	**Cap**
Nutrients to encourage	Protein	25 g	25 g	50 g	50 g
	Dietary fiber	8 g	8 g	13 g	13 g
	Calcium	600 mg	600 mg	750 mg	750 mg
	Iron	5.5 mg	5.5 mg	12 mg	12 mg
	Vitamin D	4.0 μg	4.0 μg	8.0 μg	8.0 μg
Nutrients to limit	Energy	1,300 kcal	NA	2,250 kcal	NA
	SFAs	14.4 g	NA	25.0 g	NA
	Sugar	32.5 g	NA	56.3 g	NA
	Salt equivalents	3.5 g	NA	6.0 g	NA
Food groups to encourage	Fruits	92 g	92 g	160 g	160 g
	Vegetables	161 g	161 g	280 g	280 g
	Nuts	35 g	35 g	60 g	60 g
	Legumes	46 g	46 g	80 g	80 g
	Dairy	60 g	60 g	104 g	104 g

RDV, reference daily value; NA, not applicable; SFA, saturated fatty acid.

### 2.5 Serving size for younger and older children

There are no official government-mandated serving sizes in Japan. However, serving sizes, expressed as one or more edible quantities for a single food item, are summarized in “Ordinary serving values food composition tables” (61). Serving sizes for children were calculated by applying energy ratios to the serving sizes listed (46% for younger children and 80% for older children).

### 2.6 The WHO NP model

The WHO NP model is regarded as a critical tool for implementing restrictions on food marketing for children (9). It provides a means to distinguish the foods that are likely to be part of a healthy diet (“healthy”) from those that are not (“unhealthy”). Unhealthy foods are defined as those that may contribute to an excessive intake of energy, SFAs, transfats, sugar, and salt. The WHO NP model sets thresholds for total fat, total sugar, added sugar, non-sugar sweeteners (NSS), energy, SFAs, and sodium. Each food category had different thresholds, and foods that exceeded these thresholds were classified as unhealthy. For categories 1 (chocolate and sugar confectionery, energy bars, sweet toppings, and desserts), 2 (cakes, sweet biscuits and pastries, and other sweet bakery products and dry mixes), and 4c (energy drinks, tea, and coffee), no thresholds were set, as none of the foods in these categories should be marketed to children. Therefore, in this study, these foods were considered unhealthy. We classified foods from the Japanese Food Standard Composition Table (62) into food categories based on the WHO NP model, using the category names, examples, and customs tariff codes, provided in the WHO NP guidelines, and determined whether each food was healthy, according to the thresholds.

### 2.7 Nutrient composition database

The Japanese Food Standard Composition Table 2020 Edition (8th edition), published by the Ministry of Education, Culture, Sports, Science, and Technology, Japan (62), lists 2,478 food items. Energy and nutrient content are expressed per 100 g. After excluding prepared meals, a total of 2,428 food items were obtained. Many of these items had missing data, particularly regarding total sugar content. Following previous studies (63–65), we set the sugar content to zero in raw or minimally processed seafood and meat. Food items with missing data in other categories were excluded, as were food items for which no portion sizes (edible quantities) were available, leading to an analytical sample of 1,091 food items. Meiji NPS scores per serving size for each food item were determined using the median Meiji NPS scores per serving size. The food composition table does not include data on the contents of the food groups to encourage. We set the food groups to encourage to 100% based on food category names, following previous methods (25, 26). For food items with specific fruit proportions, we adhered to the indicated ratios.

### 2.8 Validation and statistical analysis

The medians and interquartile ranges (IQRs) are used to express the Meiji NPS scores. The convergent validity of the Meiji NPS for both children per 100 g was tested using the WHO NP model and NRF9.3. The WHO NP model classifies foods as healthy or unhealthy based on the following content per 100 g of nutrients to limit: total fat, SFAs, total sugars, added sugars, NSS, and sodium. Energy was also included in the analysis. The Japanese Food Standard Composition Table (62) does not include data on added sugars or NSS. For food categories with set thresholds for added sugars or NSS in the WHO NP model and those without thresholds for total sugars, we used the stricter thresholds for total sugars. The 1,091 food items were classified as either healthy or unhealthy according to the WHO NP model. The food items classified into the healthy and unhealthy groups are presented using boxplots or histograms for all the food categories in the food composition table, as well as each food category. The differences in the Meiji NPS scores for children between the WHO unhealthy and healthy food groups were assessed using the Wilcoxon test. Spearman's correlation test was used to compare the Meiji NPS scores (per both 100 g and serving size) along with the NRF9.3 scores. All statistical analyses were conducted using the R software version 4.3.1 (The R Foundation for Statistical Computing, Vienna, Austria). A *p*-value of < 0.05 was considered statistically significant.

## 3 Results

### 3.1 The Meiji NPS for children per 100 g and per serving size

The Meiji NPS scores for the children were calculated for each of the 1,091 food items with complete nutritional information (Table 2). The Meiji NPS scores per 100 g for younger children ranged from −442.3 to 423.9 and for older children from −265.5 to 386.5. The median Meiji NPS scores for younger and older children were 81.4 and 46.7, respectively. No food items were classified as sugars and sweeteners, owing to the lack of nutrient data. Thus, the central tendency of this food category was not available.

**Table 2:** Meiji NPS scores for younger and older children per 100 g by food category.

**Item**	***n***	**Meiji NPS for younger children**				**Meiji NPS for older children**			
		**Median**	**Max**	**Min**	**IQR**	**Median**	**Max**	**Min**	**IQR**
Beverages	2	342.6	404.6	280.6	311.6 to 373.6	311.6	335.0	288.2	299.9 to 323.3
Pulses	42	263.5	385.7	59.2	160.0 to 353.3	194.5	337.9	74.3	132.4 to 279.8
Nuts and seeds	22	222.0	387.0	−175.5	190.2 to 287.1	173.8	386.5	−24.4	151.0 to 218.2
Algae	9	204.2	345.0	−253.8	61.4 to 208.2	200.7	310.4	−75.3	130.3 to 244.2
Mushrooms	24	157.2	423.9	−10.7	124.0 to 258.9	88.4	380.4	−7.8	70.4 to 202.8
Fish and seafood	350	124.4	352.9	−369.3	68.0 to 169.8	83.5	326.3	−194.4	36.4 to 130.7
Vegetables	123	106.1	305.5	10.8	87.2 to 147.9	61.7	193.8	6.9	50.9 to 84.6
Fruits	62	80.1	168.0	−354.9	67.1 to 96.7	51.7	105.9	−203.1	43.5 to 61.3
Eggs	13	66.9	182.3	−22.0	22.3 to 130.9	29.5	137.2	−18.6	9.9 to 62.3
Milk and milk products	37	64.6	238.2	−305.8	−61.0 to 108.7	84.5	227.1	−173.4	−36.2 to 107.6
Potatoes and starches	18	38.2	128.3	−13.8	27.7 to 51.5	22.1	71.8	−8.8	16.0 to 30.0
Meat	210	33.9	231.3	−254.3	−23.5 to 80.9	12.7	147.9	−149.3	−21.0 to 38.4
Cereals	77	18.1	227.9	−131.7	0.7 to 46.2	7.8	202.2	−77.1	−0.1 to 25.1
Confectionery	82	−65.7	99.8	−327.6	−114.0 to −24.2	−41.6	60.1	−189.5	−67.2 to −16.2
Seasonings and spices	16	−152.9	335.2	−442.3	−221.7 to 10.3	−90.8	289.8	−265.5	−133.4 to 4.6
Fats and oils	4	−288.4	−147.7	−435.7	−400.9 to −177.6	−168.6	−42.9	−253.0	−233.3 to −93.6
Total	1091	81.4	423.9	−442.3	13.1 to 142.5	46.7	386.5	−265.5	5.4 to 99.0

n, number of food items; Max, maximum; Min, minimum; IQR, interquartile range; NA, not applicable.

The Meiji NPS algorithm for younger and older children was used to calculate scores per serving size (Supplementary Table 1). Spearman's correlation coefficients between Meiji NPS per 100 g and NRF9.3 showed strong correlations of 0.734 and 0.733 for younger and older children, respectively (Supplementary Table 2) (66). An additional comparison between the Meiji NPS per serving size and NRF9.3 showed moderate correlations.

### 3.2 Comparing Meiji NPS for children to the WHO NP model by age group

Table 3 presents differences in Meiji NPS scores for foods classified as healthy or unhealthy based on the WHO NP model. The medians, means, standard deviations (SDs), and confidence intervals are provided for younger and older children. Significant differences were observed in Meiji NPS scores between foods classified as healthy and those classified as unhealthy by the WHO NP model. For younger children, the mean Meiji NPS scores for these two classes were 116.2 (healthy) and 21.8 (unhealthy; *p* < 0.001). Their median scores differed significantly (*p* < 0.001) between the two classes at 99.1 (healthy) and 14.4 (unhealthy). For older children, the mean Meiji NPS scores were 71.7 (healthy) and 29.5 (unhealthy) and the median Meiji NPS scores were 54.8 (healthy) and 5.6 (unhealthy), with a significant difference observed between the two groups (*p* < 0.001).

**Table 3:** Meiji NPS scores for foods classified by the WHO NP model as healthy and unhealthy.

**Meiji NPS model**	**Younger children (3–5 years)**	**Older children (6–11 yeas)**
**WHO healthy foods (*****n*** = **516)**		
Median	99.1	54.8
Mean ± SD	116.2 ± 72.3	71.7 ± 57.4
95% CI	[110.3, 122.2]	[67.0, 76.4]
**WHO unhealthy foods (*****n*** = **575)**		
Median	14.4	5.6
Mean ± SD	21.6 ± 140.1	29.5 ± 105.4
95% CI	[9.7, 34.0]	[20.4, 38.6]
*p*-Value	<0.001	<0.001

NPS, Nutritional Profiling System; SD, standard deviation; CI, confidence interval.

**Figure 1:**
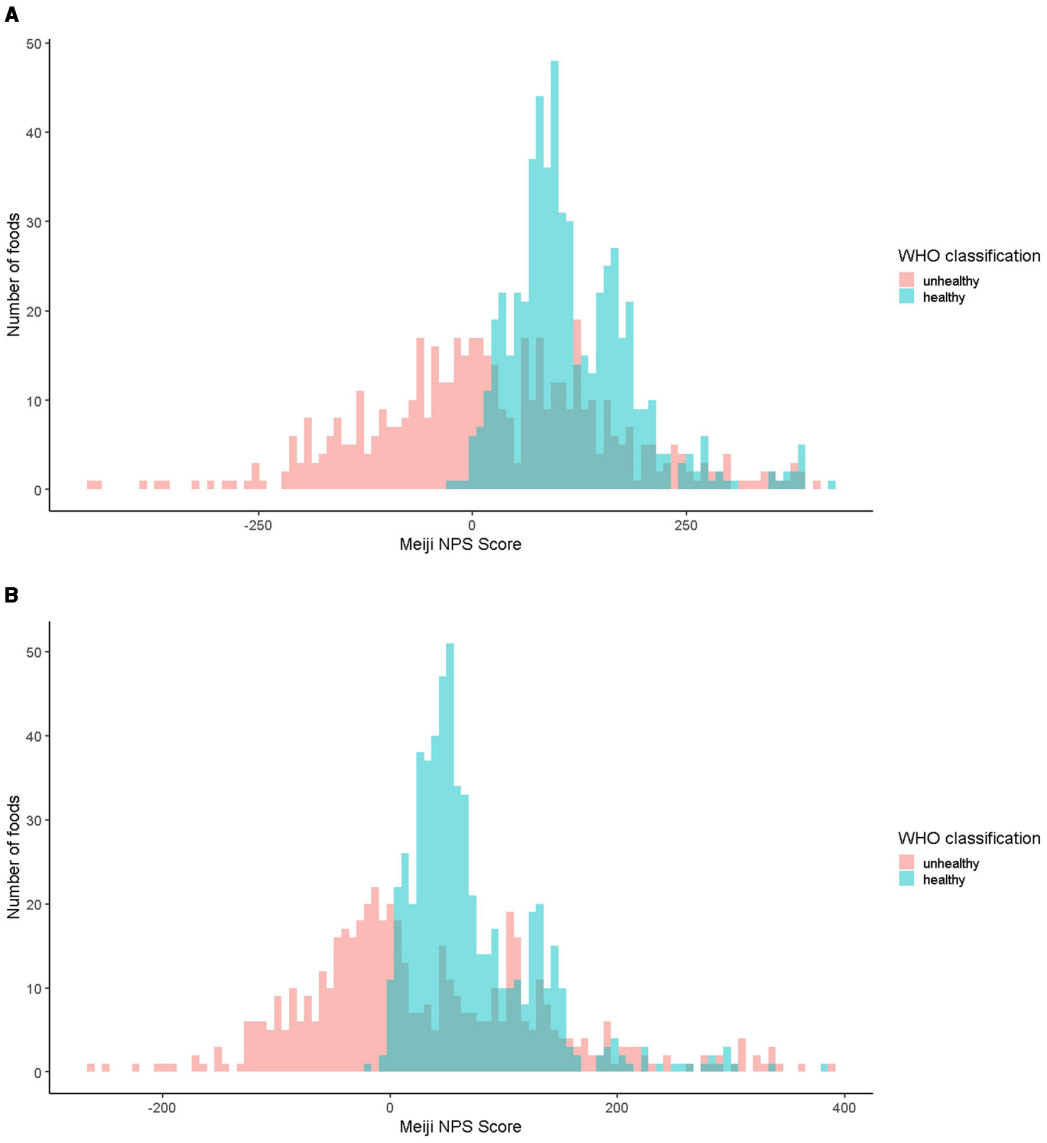
**(A)** Distribution of the Meiji NPS scores for younger children (histogram). **(B)** Distribution of the Meiji NPS scores for older children (histogram). Red indicates food items classified as unhealthy and blue as healthy. NPS, Nutritional Profiling System.

Figures 1A, B show the distribution (histogram) of the Meiji NPS scores for foods classified by the WHO NP model as healthy and unhealthy, respectively, for younger (Figure 1A) and older children (Figure 1B). For younger children, the minimum Meiji NPS scores for healthy and unhealthy food items were −22.0 and −442.3, respectively. For older children, these minimum Meiji NPS scores were −18.6 (healthy) and −265.5 (unhealthy).

### 3.3 Comparing Meiji NPS to the WHO NP model by age group and food category

**Figure 2:**
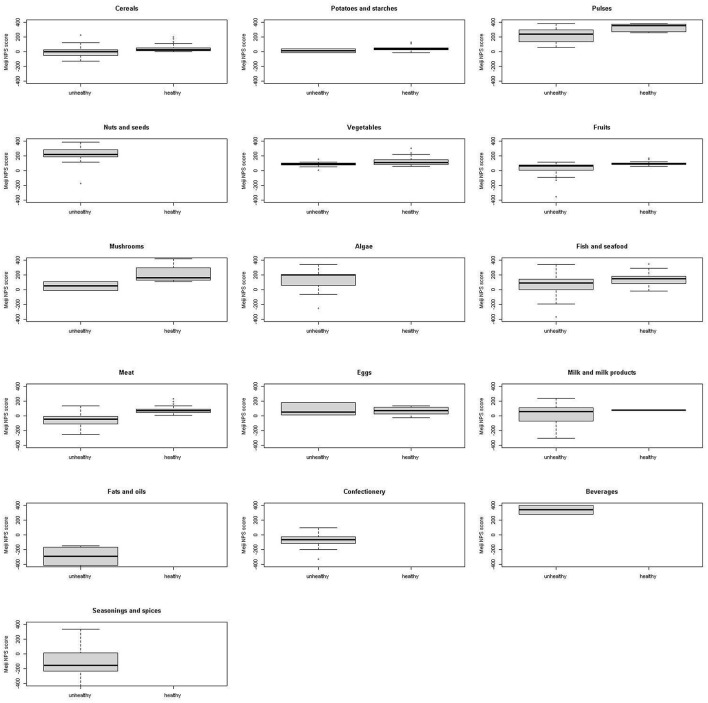
Meiji NPS (younger children) scores for foods classified by the WHO NP model as healthy or unhealthy by food category. Shown on the left of each graph are Meiji NPS scores for younger children for food items classified as unhealthy by the WHO NP model, while on the right are Meiji NPS scores for foods classified by the WHO NP model as healthy. No food items including nuts and seeds, algae, fats and oils, confectionery, and beverages were classified as healthy by the WHO NP model. NPS, Nutritional Profiling System.

**Figure 3:**
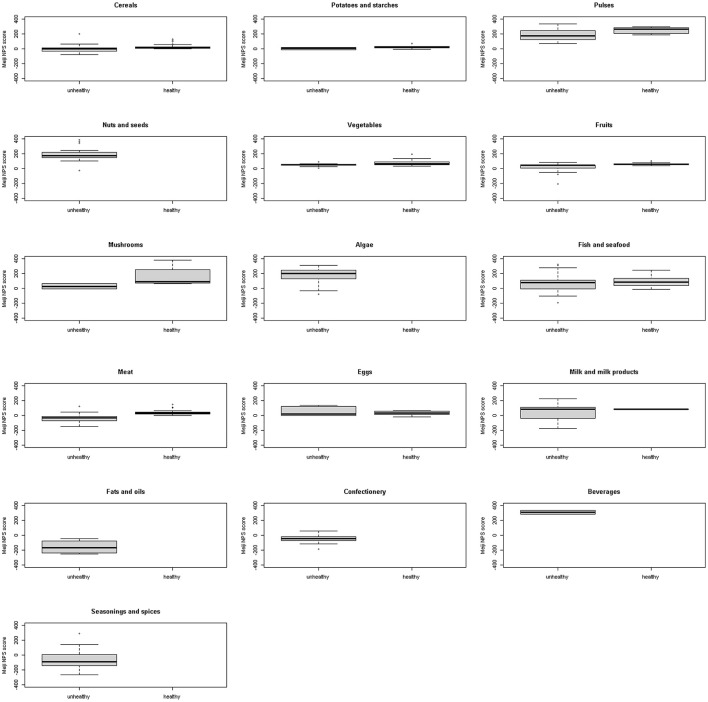
Meiji NPS (older children) scores for foods classified by the WHO NP model as healthy or unhealthy by food category. Shown on the left of each graph are NPS scores for foods classified by the WHO NP model as unhealthy and on the right MPS scores for foods classified by the WHO NP model as healthy. No food items including nuts and seeds, algae, fats and oils, confectionery, and beverages were classified as healthy by the WHO NP model. NPS, Nutritional Profiling System.

Figures 2, 3 show a comparison of the Meiji NPS for children with the WHO NP model by food category. Significant differences were observed in cereals, pulse vegetables, fruits, mushrooms, fish and seafood, and meat in the Meiji NPS for children (*p* < 0.05).

### 3.4 Comparing Meiji NPS for younger and older children to the NRF9.3 NP model

**Figure 4:**
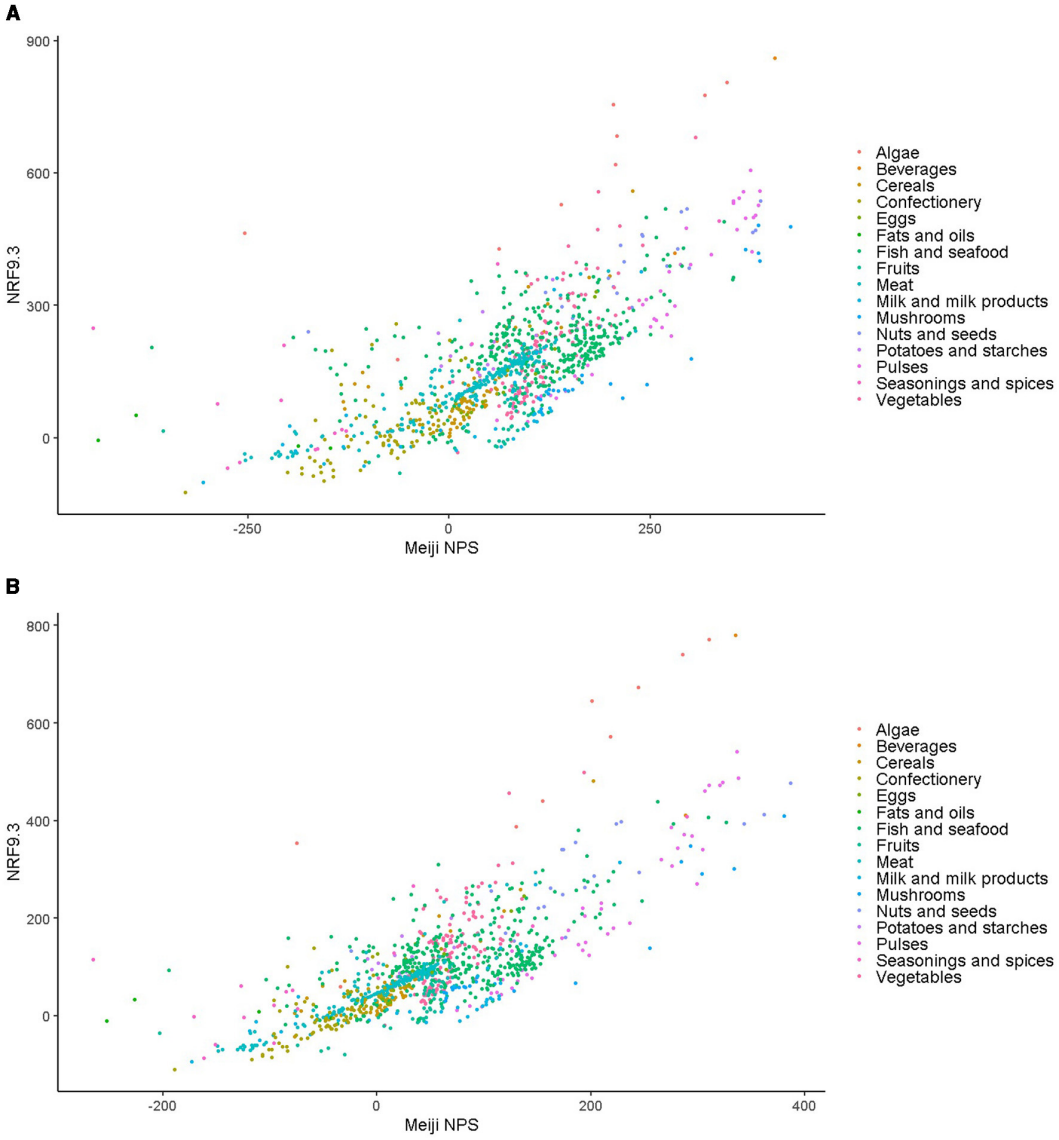
**(A)** Scatter plot of Meiji NPS and NRF9.3 scores for the same foods by age. Data are for the Meiji NPS model for younger children. **(B)** Scatter plot of Meiji NPS and NRF9.3 scores for the same foods by age. Data are for the Meiji NPS model for older children. NPS, nutritional profiling system; NRF9.3, Nutrient-Rich Foods Index 9.3.

Figure 4 shows the scatter plots illustrating the correlation between the Meiji NPS for children and NRF9.3 scores, with data shown for both younger (Figure 4A) and older children (Figure 4B). Spearman's correlation coefficients for the Meiji NPS and NRF9.3 were 0.73 for both age groups (*p* < 0.001).

## 4 Discussion

NP models, intended as the basis for FOP labels at the point of purchase, rarely address the ages of potential consumers. NP models need to address the nutritional needs of specific age groups if they are to serve as a useful benchmark for product reformulation. The first Meiji NPS for adults addressed the nutritional needs of adults and older adults (25). The present Meiji NPS for younger and older children incorporates nutrients essential for childhood development and growth. To our knowledge, this study is the first to assess the nutritional value of foods listed in the Japan Nutrient Composition Table specifically in the context of childhood nutrition. Two versions of the Meiji NPS were developed for younger and older children, with nutrient density calculated per 100 g and per serving size.

Continuous Meiji NPS scores for 1,091 foods were compared to the WHO food classification system, which categorizes foods as healthy and unhealthy. First, foods classified by the WHO NP model as healthy had significantly higher mean Meiji NPS scores than those classified as unhealthy. Based on the Meiji NPS, foods scoring higher than −22.0 for younger children or −18.6 for older children were more likely to align with the WHO's healthy classification (Figures 2, 4). However, there were major differences by food category. Nuts and seeds, algae, and beverages were classified by the WHO NP model as unhealthy. In contrast, nuts and seeds, algae, and beverages had above-median scores in both the Meiji NPS models (per 100 g). The Meiji NPS for older children produced above-median scores for milk and milk products. Dietary patterns that include milk and dairy products have beneficial effects on children's growth and development (67, 68) and lifestyle-related diseases (69–74). Pulses, vegetables, mushrooms, and fish and seafood received scores above the median Meiji NPS.

These discrepancies may be explained by differences in the composition of food groups. The Japanese food composition tables may be atypical. For example, the beverage category was composed of cocoa and green tea, both of which received high Meiji NPS scores. However, neither scoring system considered caffeine. One potential concern is that excessive caffeine intake can adversely affect the health, growth, and development of children (75–78). Similarly, the categories of nuts and seeds, algae, seasonings, and spices, classified as unhealthy by the WHO NP model, received high scores on the Meiji NPS. The consumption of nuts and seeds and algae has beneficial effects on growth and development, as well as on the risk of lifestyle-related diseases (70, 79, 80).

The basis of the calculations, 100 g or the serving size, affected the Meiji NPS scores. While using 100 g as reference quantity allows for a cross-sectional assessment, nutrient density calculated per serving size may be more comprehensible to the consumer (81). In the Meiji NPS for younger children, dairy and fruits scored below the median when calculated per 100 g but above the median when calculated per serving size. Dairy products and fruits are beneficial for the growth and development of children (67, 68, 82–84). The ability to use both evaluation methods for younger and older children is a strength of the Meiji NPS, allowing flexibility, depending on the context. Therefore, an evaluation that considers both per 100 g and serving size may be important.

This study has some limitations. First, not all food items were evaluated using the Meiji NPS for younger and older children because of missing data in the 2020 Japanese Food Standard Composition Table. Second, the book “*Ordinary serving values food composition tables*” (61) was used as reference for serving sizes in Japan. However, the serving sizes in the book do not reflect those of younger or older children. Instead, the study estimated serving sizes for younger and older children by adjusting adult serving sizes based on the energy ratios. Finally, an examination of the predictive validity is necessary to determine whether diets associated with high scores on the Meiji NPS for younger and older children are associated with better growth and development.

## 5 Conclusion

The new NPS for Japanese children was developed based on the Meiji NPS. Convergent validity of the Meiji NPS was confirmed by comparison with the WHO NP model and NRF9.3. However, some differences emerged between the Meiji NPS and the WHO NP model. The WHO NP model identified specific food groups that are subject to restriction on advertising. For example, the WHO NP model does not permit the marketing of children's food items classified as unhealthy, including nuts and seeds, algae, fats and oils, confectioneries, beverages, and seasonings and spices. However, this approach may not effectively support product reformulation efforts in Japan. In contrast, the Meiji NPS may be better suited to addressing the health needs of younger and older children in Japan. The NPS may also support Japanese food manufacturers in producing healthier products for children.

## Data Availability

The food composition data presented in this study are openly available in the Japanese Food Standard Composition Table 2020 Edition (8th Edition) at https://www.mext.go.jp/a_menu/syokuhinseibun/mext_01110.html. The ordinary serving values data presented in this study are available in the book “[Ordinary serving values food composition tables] jouyouryou shokuhin seibun hayami hyou (in Japanese)” published by Ishiyaku Publishers, Inc.
