# Correction: Development and validation of the Meiji Nutritional Profiling System for children

**DOI:** 10.3389/fnut.2025.1650235

**Published:** 2025-07-09

**Authors:** Frontiers Production Office

**Affiliations:** Frontiers Media SA, Lausanne, Switzerland

**Keywords:** nutrient profiling, World Health Organization, nutrient-rich food index, growth, development, children, Japanese diet

There was a mistake in Table 1 as published. The values for SFAs and sugar were reversed under the heading RDV in both categories. The corrected Table 1 appears below.

**Table 1:** RDVs and caps of the Meiji NPS for younger children and older children.

**Items**		**Meiji NPS for younger children**		**Meiji NPS for older children**	
		**RDV**	**Cap**	**RDV**	**Cap**
Nutrients to encourage	Protein	25 g	25 g	50 g	50 g
	Dietary fiber	8 g	8 g	13 g	13 g
	Calcium	600 mg	600 mg	750 mg	750 mg
	Iron	5.5 mg	5.5 mg	12 mg	12 mg
	Vitamin D	4.0 μg	4.0 μg	8.0 μg	8.0 μg
Nutrients to limit	Energy	1,300 kcal	NA	2,250 kcal	NA
	SFAs	14.4 g	NA	25.0 g	NA
	Sugar	32.5 g	NA	56.3 g	NA
	Salt equivalents	3.5 g	NA	6.0 g	NA
Food groups to encourage	Fruits	92 g	92 g	160 g	160 g
	Vegetables	161 g	161 g	280 g	280 g
	Nuts	35 g	35 g	60 g	60 g
	Legumes	46 g	46 g	80 g	80 g
	Dairy	60 g	60 g	104 g	104 g

RDV, reference daily value; NA, not applicable; SFA, saturated fatty acid.

The original version of this article has been updated.

