# Supplementary material for: Development and validation of the Meiji Nutritional Profiling System for children
Source: Front Nutr. 2025 Jun 17;12:1611286. doi: 10.3389/fnut.2025.1611286 (PMC12209353; doi:10.3389/fnut.2025.1611286)
Supplement: Supplementary file 1 [file Table_1.docx]

Supplementary Material

Supplementary Table 1 The Meiji NPS scores for younger and older children per serving size

| Item | n | Younger children | | | | Older children | | | |
| --- | --- | --- | --- | --- | --- | --- | --- | --- | --- |
|  |  | Median | Max | Min | IQR | Median | Max | Min | IQR |
| Cereals | 77 | 3.4 | 45.7 | -48.5 | 0.7 to 17.0 | 2.7 | 42.0 | -49.2 | -0.1 to 14.5 |
| Potatoes and starches | 18 | 8.9 | 27.4 | -3.5 | 5.9 to 12.3 | 9.0 | 28.7 | -2.9 | 5.6 to 12.2 |
| Sugars and sweeteners | 0 | NA | NA | NA | NA | NA | NA | NA | NA |
| Pulses | 42 | 57.6 | 126.7 | 7.0 | 41.3 to 73.2 | 56.6 | 122.5 | 6.9 | 40.6 to 72.7 |
| Nuts and seeds | 22 | 27.3 | 48.1 | 3.0 | 10.7 to 33.7 | 27.5 | 48.0 | 3.0 | 10.6 to 32.1 |
| Vegetables | 123 | 14.4 | 49.6 | 0.4 | 8.1 to 21.6 | 14.5 | 49.1 | 0.4 | 8.2 to 21.7 |
| Fruits | 62 | 22.3 | 73.0 | -25.2 | 10.5 to 28.2 | 22.5 | 72.9 | -26.8 | 10.4 to 28.1 |
| Mushrooms | 24 | 11.1 | 64.1 | -0.5 | 8.4 to 14.7 | 10.7 | 59.2 | -0.6 | 8.2 to 14.3 |
| Algae | 9 | 7.0 | 31.6 | -2.2 | 4.3 to 16.2 | 6.8 | 27.9 | -2.1 | 3.8 to 18.4 |
| Fish and seafood | 350 | 28.6 | 138.2 | -35.3 | 8.9 to 60.8 | 24.1 | 125.7 | -38.9 | 7.4 to 52.9 |
| Meat | 210 | 6.7 | 38.6 | -52.6 | -2.7 to 14.7 | 3.9 | 31.2 | -53.7 | -4.3 to 11.8 |
| Eggs | 13 | 9.6 | 35.2 | -1.0 | 3.1 to 13.9 | 6.1 | 29.9 | -1.5 | 1.7 to 11.6 |
| Milk and milk products | 37 | 12.9 | 121.3 | -14.3 | -2.1 to 71.5 | 13.9 | 125.0 | -14.3 | -2.2 to 73.1 |
| Fats and oils | 4 | -13.5 | 1.3 | -23.0 | -21.2 to -4.5 | -13.7 | -0.4 | -23.3 | -21.5 to -5.1 |
| Confectionery | 82 | -8.6 | 16.3 | -36.5 | -15.4 to -3.6 | -9.3 | 14.4 | -38.4 | -16.7 to -4.1 |
| Beverages | 2 | 20.8 | 34.6 | 7.0 | 13.9 to 27.7 | 20.0 | 33.1 | 6.9 | 13.4 to 26.5 |
| Seasonings and spices | 16 | -7.4 | 3.8 | -25.4 | -12.0 to 0.5 | -7.6 | 3.6 | -26.5 | -12.2 to 0.4 |
| Total | 1091 | 12.2 | 138.2 | -52.6 | 2.2 to 28.7 | 10.9 | 125.7 | -53.7 | 1.2 to 27.3 |

NPS, Nutrient Profiling System; n, number of food items; Max, maximum; Min, minimum; IQR, interquartile range; NA, not applicable.

Supplementary Table 2 Spearman’s correlation coefficients between the Meiji NPS and the NRF9.3

|  | 100 g | | | | Serving size | | | |
| --- | --- | --- | --- | --- | --- | --- | --- | --- |
|  | Younger children | | Older children | | Younger children | | Older children | |
|  | r | p-value | r | p-value | r | p-value | r | p-value |
| Cereals | 0.700 | <0.001 | 0.796 | <0.001 | 0.425 | <0.001 | 0.547 | <0.001 |
| Potatoes and starches | 0.307 | 0.216 | 0.358 | 0.145 | 0.020 | 0.941 | 0.077 | 0.761 |
| Sugars and sweeteners | NA | NA | NA | NA | NA | NA | NA | NA |
| Pulses | 0.946 | <0.001 | 0.962 | <0.001 | -0.217 | 0.167 | -0.204 | 0.195 |
| Nuts and seeds | 0.839 | <0.001 | 0.843 | <0.001 | -0.082 | 0.717 | -0.171 | 0.445 |
| Vegetables | 0.769 | <0.001 | 0.657 | <0.001 | 0.070 | 0.441 | 0.043 | 0.639 |
| Fruits | 0.705 | <0.001 | 0.784 | <0.001 | 0.168 | 0.190 | 0.216 | 0.092 |
| Mushrooms | 0.884 | <0.001 | 0.910 | <0.001 | 0.570 | 0.004 | 0.614 | 0.002 |
| Algae | 0.900 | 0.002 | 0.967 | <0.001 | 0.167 | 0.678 | 0.267 | 0.493 |
| Fish and seafood | 0.422 | <0.001 | 0.451 | <0.001 | 0.229 | <0.001 | 0.224 | <0.001 |
| Meat | 0.929 | <0.001 | 0.894 | <0.001 | 0.931 | <0.001 | 0.880 | <0.001 |
| Eggs | 0.802 | 0.002 | 0.687 | 0.012 | 0.637 | 0.022 | 0.604 | 0.032 |
| Milk and milk products | 0.750 | <0.001 | 0.882 | <0.001 | 0.409 | 0.013 | 0.456 | 0.005 |
| Fats and oils | -0.800 | 0.333 | 0.200 | 0.917 | -0.800 | 0.333 | 0.200 | 0.917 |
| Confectionery | 0.653 | <0.001 | 0.734 | <0.001 | 0.575 | <0.001 | 0.626 | <0.001 |
| Beverages | 1.000 | 1.000 | 1.000 | 1.000 | -1.000 | 1.000 | -1.000 | 1.000 |
| Seasonings and spices | 0.532 | 0.036 | 0.685 | 0.004 | 0.532 | 0.036 | 0.709 | 0.003 |
| Total | 0.734 | <0.001 | 0.733 | <0.001 | 0.521 | <0.001 | 0.526 | <0.001 |

NPS, Nutrient Profiling System; NRF9.3, Nutrient-Rich Foods Index 9.3; NA, not available.
